# Psychometric evaluation of the Affiliate Stigma Scale for Asian Indian dementia family caregivers living in the United States

**DOI:** 10.1002/bsa3.70047

**Published:** 2025-11-07

**Authors:** Anju Wadhawan, Rula Btoush, Peijia Zha, Olga F. Jarrín

**Affiliations:** 1 Division of Nursing Science, Rutgers Health School of Nursing, Rutgers, The State University of New Jersey, Newark, New Jersey, USA; 2 Herbert and Jacqueline Krieger Klein Alzheimer’s Research Center, Rutgers Brain Health Institute, Rutgers, The State University of New Jersey, New Brunswick, New Jersey, USA; 3 Community Health and Aging Outcomes Lab, Rutgers Institute for Health, Health Care Policy and Aging Research, Rutgers, The State University of New Jersey, New Brunswick, New Jersey, USA; 4 Center for Health Equity and Systems Research, Rutgers Health School of Nursing, Rutgers, The State University of New Jersey, Newark, New Jersey, USA

**Keywords:** adult and older adults, chronic illness, family, immigrant health, mental health, quantitative, social determinants of health

## Abstract

**Background:**

The Affiliate Stigma Scale measures the internalized stigma experienced by caregivers. Among Asian Indians, having a family member with dementia is sometimes perceived as punishment for past-life sins, underscoring the need for culturally relevant tools for measuring stigma. This study evaluated a shortened, culturally adapted version of the Affiliate Stigma Scale for Asian Indian dementia family caregivers in the United States.

**Methods:**

Surveys were completed by 222 caregivers, recruited through community and social networks. Analyses included descriptive statistics, reliability testing, exploratory and confirmatory factor analysis, and construct validity.

**Results:**

The original 22-item scale showed poor model fit. Analyses supported a unidimensional 11-item version with good fit indices and excellent reliability. Known-groups validity was supported, with higher stigma scores among female versus male caregivers and among caregivers of South versus North Indian origin.

**Conclusion:**

This culturally adapted 11-item version is a valid and reliable instrument to assess affiliate stigma in Asian Indian American dementia family caregivers.

## INTRODUCTION

The rising prevalence of dementia in the United States poses a significant burden on society, with unpaid caregiving costs exceeding $339 billion annually.^1^ Among Medicare beneficiaries who died in 2019, 43% had a diagnosis of dementia, including 41% of Asian American and Pacific Islander beneficiaries.^2^ Asian Americans face unique challenges in dementia care, including lower rates of diagnosis, delayed treatment,^3^ and longer survival after diagnosis.^4^ These patterns are linked to stigma, language barriers, a shortage of bilingual healthcare providers, and a lack of culturally appropriate diagnostic tools.^5–7^ Such disparities have broad policy implications and underscore the need for culturally responsive resources.^8^ For caregivers, barriers to formal support are compounded by stress, stigma, and financial strain, particularly among women who reduce or leave workforce participation.^1,9^ Within this context, the culturally and linguistically diverse Asian Indian population, now the largest Asian immigrant subgroup,^10^ faces distinctive challenges related to caregiving.^11–17^

These challenges are shaped by collectivist values and filial piety, which frame caregiving as a familial duty rather than one shared with formal systems.^13,14^ Reliance on kinship networks, while protective, may intensify caregiver burden under financial constraints and limited culturally competent services.^12^ Social isolation, language barriers, and the preservation of cultural identity further contribute to hidden caregiving struggles.^14^ Within these contexts, dementia-related stigma often persists across generations, reinforced by cultural taboos.^15,17^ Without culturally responsive education and interventions, such stigma heightens caregiver stress, delays help seeking, and worsens long-term health outcomes.^13,16^

Affiliate stigma refers to the internalized shame, guilt, and devaluation experienced by family caregivers due to their association with a stigmatized individual, such as a person with dementia.^18,19^ Unlike public stigma, which targets the individual with the illness,^19,20^ affiliate stigma emphasizes the impact of societal bias on caregivers, often leading to psychological distress, social withdrawal, and reduced use of support services.^21–24^ In dementia caregiving, affiliate stigma is amplified by cultural norms.^24–26^ In Asian Indian culture, caregiving is viewed as a sacred duty grounded in filial piety and intergenerational co-residence.^14^ However, when symptoms of dementia are interpreted as spiritual failings or divine punishment, caregivers – particularly women and in-laws – may experience heightened stigma.^24–27^

The Affiliate Stigma Scale was developed in Hong Kong for caregivers of individuals with intellectual disabilities and mental illness.^17^ It has since been adapted across multiple cultural and clinical contexts, including dementia caregivers in Taiwan and Iran,^25,28^ and translated into various languages, including Taiwanese, Hindi, Persian, Greek, Malay, Turkish, and Persian.^28–34^ However, these adaptations have largely been developed in East Asian or non-immigrant contexts and may not capture the sociocultural dynamics shaping dementia-related stigma among Asian Indian caregivers in the United States. In this population, collectivist family structures, gendered caregiving expectations, and religious attributions of illness can profoundly alter how stigma is experienced and expressed. For example, caregiving roles often fall to daughters-in-law, who may experience stigma differently from spouses or adult children, while beliefs linking dementia to past-life deeds or insufficient devotion can intensify shame and concealment.^12,23,35,36^ Existing instruments that do not reflect these culturally specific beliefs and roles risk underestimating stigma, misclassifying its dimensions, and obscuring targets for intervention.

This study addresses this gap by evaluating the reliability and validity of a culturally adapted Affiliate Stigma Scale specifically for Asian Indian dementia family caregivers in the United States, which accurately reflects the cultural norms and stigma expressions of this community. The goal is to provide an appropriate measure to advance research and interventions in this population.^12,23,35,36^

### 1 | METHODOLOGY

#### 1.1 | Affiliate Stigma Scale background and adaptation

The Affiliate Stigma Scale is a 22-item instrument originally developed in Hong Kong to measure internalized stigma among family caregivers of individuals with intellectual disabilities and mental illness.^17^ Grounded in cognitive behavioral theory, it conceptualizes affiliate stigma as three interrelated dimensions—cognitive (internalized negative beliefs), affective (emotional responses such as shame and guilt), and behavioral (avoidance and withdrawal)—and has demonstrated excellent psychometric properties across diverse populations (Cronbach’s *α* = 0.88 to 0.96).^17,20,29–33^

The first author obtained permission from the original developers^17^ to adapt the Affiliate Stigma Scale for Asian Indian dementia family caregivers in the United States. The adaptation process focused on cultural congruence for this target population. A panel of eight Asian Indian American individuals reviewed the original 22-item Affiliate Stigma Scale for clarity, relevance, and cultural appropriateness, suggesting revisions to improve cultural fit (e.g., modifying “I lose face” to “I feel ashamed”). Four subject matter experts in gerontological nursing and caregiver research then evaluated the revised items for alignment with the intended construct. Finally, a diverse panel of Asian Indian community members, including both former dementia caregivers and non-caregivers, reviewed the adapted items in a structured session, confirming the cultural and conceptual relevance of the adapted Affiliate Stigma Scale for this population.

### 1.2 | Participants and sampling plan

A total of 222 Asian Indian family caregivers of individuals living with dementia in the United States participated. The sample size was based on the guideline of having at least 10 participants per item for factor analysis (22 items; *n* = 222).^37^ Participants were recruited from July to October 2024 through personal and professional networks, social media (LinkedIn, Facebook, WhatsApp, and Instagram), and flyers posted at Asian Indian community centers, including senior centers, ethnic grocery stores, Ayurvedic clinics, neurology clinics, cultural events, and religious institutions (Gurdwaras and Hindu temples). A copy of the recruitment flyer is included as supplemental content. Eligibility criteria included self-identification as an Asian Indian dementia family caregiver, age 18 or older, residence in the United States, and the ability to read English. Participants could complete the survey online (via Qualtrics, with bot prevention and automated screening for fraudulent responses) or on paper (with in-person eligibility screening by research staff). All participants provided informed consent.

### 1.3 | Measures

In addition to the adapted Affiliate Stigma Scale, participants reported demographics, including age, sex, marital status, education, employment, and years living in the United States, Indian state of origin, primary language spoken at home, relationship to the person with dementia, living arrangement of family member with dementia, and whether the family member had a formal dementia diagnosis. For analysis, age (<45, 45 to 55, and ≥55 years), education (any high school/trade, bachelor’s, and graduate), and state of origin (North vs South India) were recoded.

### 1.4 | Data analysis

Data were analyzed using SPSS software (version 29) and AMOS (version 29). Descriptive statistics (mean, standard deviation [SD], frequency, and proportion) were used to summarize sample characteristics and item responses. Internal consistency was assessed using item-to-total correlation coefficients (ITC; acceptable ≥ 0.30) and Cronbach’s *α* (*α* ≥ 0.70 is acceptable).^38^

The suitability of the data for factor analysis was confirmed by a Kaiser-Meyer-Olkin (KMO) value of 0.952 (>0.5 indicates adequacy) and a significant Bartlett’s Test of Sphericity (*χ*^*2*^ = 3926.385, df = 231, *p* < 0.001).^39,40^ Exploratory factor analysis (EFA) was used to evaluate the original three-factor structure (affect, behavior, and cognition) and alternate solutions.^17^ Eigenvalues > 1 and the percentage of total variance accounted for by the factors were used as the criteria to determine the number of factors for each construct of the instrument. Factor loadings of the items were estimated using the principal component method. Items with low communalities (<0.40) or cross-loadings (>0.30) were iteratively removed.

Confirmatory factor analysis (CFA) was used to assess the model for (a) the original three-factor model for 22 items, (b) a single-factor model for 22 items, and (c) a single-factor model of the final reduced set of items. Model fit indices included *χ*^*2*^/df, goodness-of-fit index (GFI), comparative fit index (CFI), Tucker–Lewis Index (TLI), root mean square error of approximation (RMSEA), and standardized root mean square residual (SRMR). The *χ*^*2*^/df less than or equal to 3 demonstrated a good model fit. GFI and CFI values close to 0.9 and both RMSEA and SRMR values < 0.08 were considered indicative of a good model fit.^41^

Finally, known-groups validity was evaluated using independent sample *t* tests and analysis of variance (ANOVA) to compare stigma scores by caregiver sex,^5,7^ region of origin,^15,22^ relationship to family member with dementia,^3^ and primary language spoken at home.^7^

## 2 | RESULTS

### 2.1 | Sample characteristics

The sample characteristics are summarized in Table 1. Of the 222 participants, slightly more than half were male, and the majority were married or in a domestic partnership (81%). In terms of age, 38% were under 45 years, 35% were 45 to 54 years, and 27% were 55 years or older. Most participants were college educated and employed full-time. Half of the participants had lived in the United States for over 20 years. Most traced their origin to Northern India. The most common languages spoken at home included Punjabi, Hindi, Gujarati, Telugu, Bengali, and Marathi.

### 2.2 | Exploratory factor analysis

**FIGURE 1:**
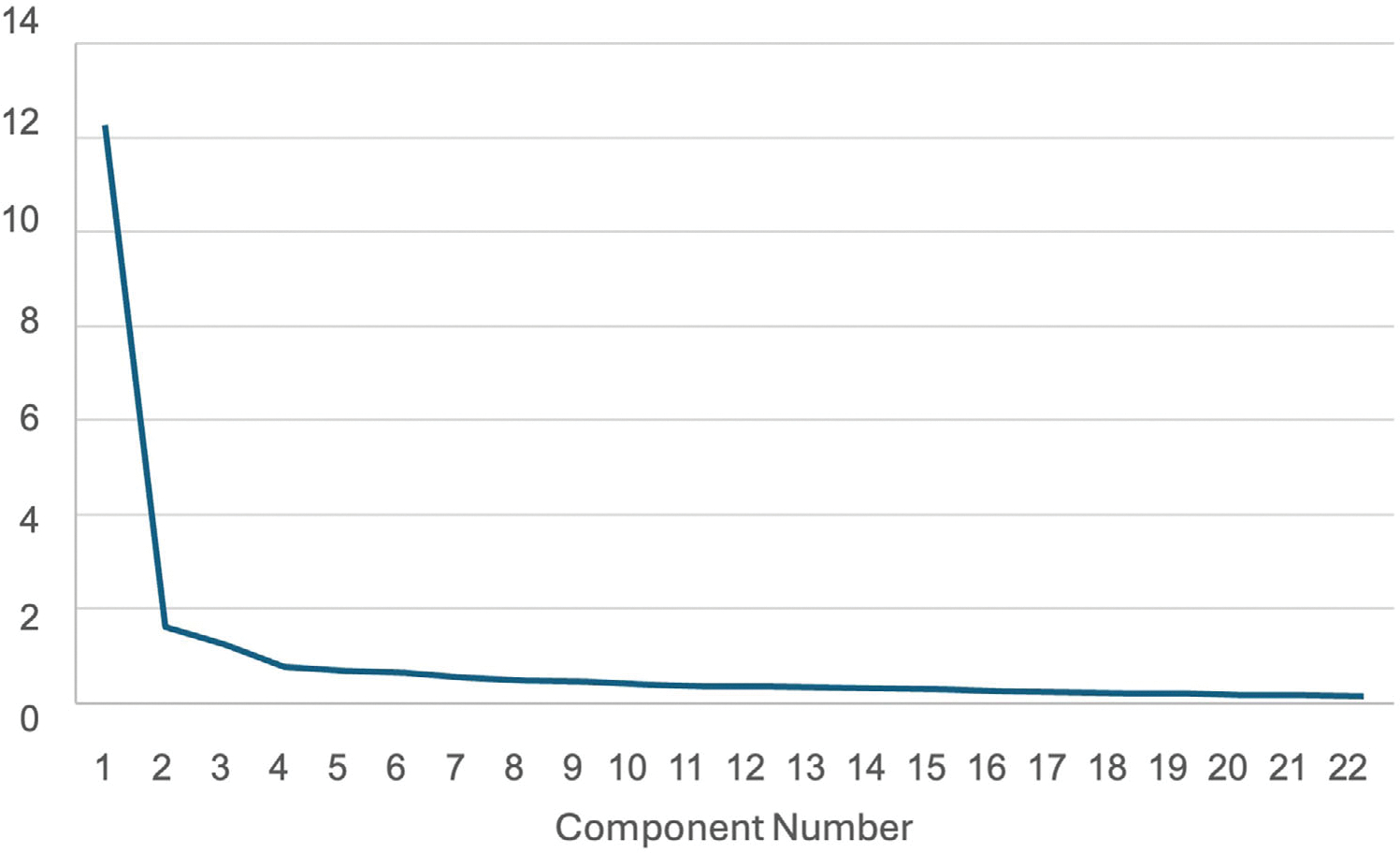
Scree plot of eigenvalues from exploratory factor analysis (EFA) of adapted 22-item Affiliate Stigma Scale illustrating a dominant first factor supporting a unidimensional solution.

The initial EFA using principal component analysis with varimax rotation method resulted in three factors with eigenvalues above 1, explaining 69% of the total variance. The scree plot (Figure 1) showed a steep drop after the first factor, followed by minor bends after the second and fourth factors. Examination of the three-factor solution revealed substantial cross-loadings and poor alignment with the original theoretical subscales of the Affiliate Stigma Scale.

Among the seven cognition items, only four had strong loadings on this factor, with the remaining items loading on the behavior subscale. Among the seven affect items, only three loaded as expected, with others cross-loading on the cognition and behavior subscales. Among the eight behavior items, four loaded as expected, with the remaining items loading on the cognition subscale.

Given these inconsistencies, a two-factor solution was examined. This model explained 63% of the variance, but a disproportionate distribution of items, with the first factor capturing 17 items across all three domains, while the second factor included only five items. These findings indicated a heavily unbalanced structure dominated by a single latent construct. Therefore, a one-factor solution was tested. The scree plot supported this approach, showing a sharp decline after the first factor with subsequent eigenvalues leveling off thereafter (Figure 1). The resulting one-factor model explained 55.81% of the variance (*λ* = 12.279), with factor loadings ranging from 0.573 to 0.832. These results support the adapted Affiliate Stigma Scale as a unidimensional measure of affiliate stigma among Asian Indian dementia caregivers.

### 2.3 | Confirmatory factor analysis

The CFA of the original three-factor, 22-item model demonstrated poor fit across all indices (Table 2), confirming that the theorized cognitive, affective, and behavioral dimensions were not valid for this population. In contrast, the CFA of the reduced 11-item one-factor model showed substantially improved fit (GFI = 0.91; CFI = 0.95; TLI = 0.95; *χ*^*2*^/df = 2.60). While the RMSEA (0.085) and SRMR (0.085) slightly exceeded conventional close-fit thresholds (RMSEA ≤ 0.08), they remained within acceptable limits. All standardized factor loadings were moderate to strong (0.57 to 0.83) (Table 3), confirming the unidimensional structure of the adapted Affiliate Stigma Scale among Asian Indian dementia caregivers.

### 2.4 | Internal consistency

The 11-item adapted Affiliate Stigma Scale demonstrated excellent internal consistency, with Cronbach’s *α* = 0.93. Corrected item-total correlations ranged from 0.591 to 0.793, indicating moderate to strong relationships between each item and the overall scale (Table 3).

### 2.5 | Known groups validity

Known-groups validity analyses supported expected differences in total scores across key demographic and contextual groups (Table 4). Significantly higher Affiliate Stigma Scale scores were observed among female caregivers compared to males (*t* = −2.762, *p* = 0.012, *d* = −0.304) and among caregivers originating from southern India compared to northern India (*t* = −2.592, *p* = 0.005, *d* = −0.403). There was a significant sex-by-region interaction, *F*(1, 217) = 7.54, *p* = 0.007, partial *η*^*2*^ = 0.034. Among participants from northern India, females scored higher than males (M = 20.3, SD = 6.2 vs M = 16.7, SD = 6.2, *d* = 0.579). Males from southern India scored substantially higher than their counterparts from northern India (M = 21.9, SD = 7.5 vs M = 16.7, SD = 6.2, *d* = 0.779), while females from northern and southern India did not differ (M = 20.3, SD = 6.2 vs M = 19.9, SD = 6.5, *d* = −0.064). Differences by family relationship to the person with dementia approached statistical significance, *F*(4, df) = 2.20, *p* = 0.070, partial *η*^*2*^ = 0.039, with nieces and nephews reporting notably lower scores (M = 14.8, SD = 6.0) compared to other family members (range M = 19.7 to 20.1). There was also a significant effect of primary language spoken at home, *F*(7, 214) = 2.21, *p* = 0.034, partial *η*^*2*^ = 0.068. Mean scores ranged from 16.3 (SD = 5.2) among Gujarati speakers to 24.3 (SD = 5.5) among Bengali speakers. No significant differences were found by age group, work status, education level, or whether the family member had a formal dementia diagnosis. Full results are included in Table S1.

## 3 | DISCUSSION

This study evaluated the psychometric properties of a culturally adapted the Affiliate Stigma Scale among Asian Indian dementia family caregivers in the United States. The results support an 11-item unidimensional scale with strong internal consistency and construct validity. Known-groups comparisons demonstrated higher affiliate stigma among women, caregivers from southern India, and those caring for relatives with a formal dementia diagnosis. A significant gender-by-region interaction further highlighted that female caregivers from northern India reported particularly high stigma, and men from southern India reported substantially higher stigma than men from northern India, underscoring the intersection of gender and regional origins in shaping stigma experiences. Caregivers who were nieces or nephews of the person with dementia reported notably lower stigma compared to other family members. These patterns align with prior research indicating gendered caregiving experiences and regional variation in cultural norms related to family reputation, honor (*izzat*), duty (*seva*), and disease disclosure in South Asian communities.^15,7,42,43^

Regional and sex/gender differences further contextualize this dynamic. Sex differences were moderately higher for females versus males in northern India but small in southern India. Regional differences were large for males from northern versus southern India and negligible for females. Northern Indian cultural frameworks often emphasize extended family involvement and public reputation, potentially intensifying stigma among women, who may bear primary responsibility for managing family honor in the context of illness. Meanwhile, elevated stigma among women from southern India may reflect differing regional gender norms and the challenges of navigating caregiving roles traditionally associated with women.^22^ These regional and gender differences mirror current findings by other researchers,^13,42^ but they may not be generalizable to all Asian Indians in the United States. In addition, generational differences are crucial: Many Asian Indians who immigrated after 1965 are now elderly individuals requiring care, and their caregivers – primarily Gen X and millennial adults – may view stigma and caregiving differently due to acculturation and evolving cultural norms.^44^ Future research should address these regional and generational differences to maintain the scale’s reliability and cultural relevance.

Caregivers’ stigma scores varied significantly by language, reflecting how culturally embedded idioms (e.g., Hindi *sathiyana* and Tamil *paittiyam*) may perpetuate dementia stigma and delay care seeking,^15^ consistent with evidence that linguistic frames shape stigma and access to services across diverse communities.^45^ Prior studies among South Asian caregivers have also documented the centrality of community perceptions and karmic beliefs in shaping both internalized and enacted stigma,^19^ supporting the conceptual validity of a unified construct in this context. These findings highlight that tools developed in Western contexts – which often separate cognitive, affective, and behavioral domains – may fail to capture the holistic and relational experience of stigma in collectivist cultures.^8,23^

### 3.1 | Comparison to prior studies

Our findings are consistent with previous reports of high stigma in South Asian dementia caregiving but provide novel evidence of interaction effects by gender and regional origin within the diaspora.^7^ The balanced gender distribution of our sample (more male caregivers than typically observed)^12^ warrants consideration and may reflect unique recruitment methods or shifting caregiving norms in immigrant communities. Future research should explore gender dynamics and caregiving role negotiations in greater depth.^11,46^ Differences from prior studies in East Asian populations (e.g., Taiwan)^25,47^ may be attributable not only to education-level differences, as noted, but also to cultural variations in filial norms, religious interpretations of illness, and access to culturally competent services.^8,23,46–48^

### 3.2 | Strengths, limitations, and future directions

Key strengths of this study include the large, diverse sample of Asian Indian caregivers and the rigorous psychometric evaluation. A notable strength of this study is the inclusion of a sample with slightly more male participants than typically observed in caregiving research. This reflects recruitment beyond female-dominated convenience samples and captures the perspectives of male caregivers, who are often underrepresented in the literature. Limitations include the reliance on self-reported measures, potential selection bias from community-based recruitment, and lack of longitudinal data to assess sensitivity to change. Non-significant group differences (e.g., age, education, and employment) may reflect the nature of the sample rather than an absence of association. Future studies should aim to validate this tool across a broader range of populations, explore how individual background factors and family responsibilities may influence caregiver experiences, and examine broader contextual elements that may contribute to stigma.^49,50^

### 3.3 | Implications for research and practice

A brief, culturally validated scale has significant implications for research, clinical screening, and program evaluation. It enables the assessment of affiliate stigma in understudied populations and can inform targeted interventions such as psychoeducation, community outreach, and culturally adapted support groups.^6,49^ It allows for the evaluation of caregiver-related perceptions and stress in groups that have not been widely examined in the existing literature. This instrument may support the design of broadly applicable educational efforts and informational resources. Longitudinal studies could investigate stigma over time, while a translated version may enhance accessibility and applicability in clinical practice settings.

## 4 | CONCLUSION

This study presents a concise and reliable measure of Asian Indian family caregiver stigma that can be used in research on dementia caregiving in the United States, offering a generalizable method for measuring stigma and enhancing our understanding of caregiver challenges. By capturing the integrated and context-specific experience of stigma – shaped by sex/gender, regional origin, and family roles – this instrument can be used to advance culturally informed research and practice. Ultimately, it contributes to more equitable dementia care and support for immigrant families.^8,48,50^

## Supplementary Material

Affiliate Stigma Scale for Dementia Family Caregivers

Recruitment Flyer

Table s1

Additional supporting information can be found online in the Supporting Information section at the end of this article.

## Figures and Tables

**TABLE 1:** Demographic characteristics of study sample (*N* = 222).

Variable	Category	*n*	Percentage valid (%)

Age	<45 years	85	38.3
	45 to 54 years	77	34.7
	≥55	60	27.0
Sex	Male	117	52.7
	Female	105	47.3
Marital status	Single (never married)	32	14.4
	Married, or in a domestic relationship	180	81.1
	Widowed	4	1.8
	Divorced/Separated	6	2.7
Education level	High school or trade school	45	18.5
	Bachelor’s	94	42.3
	Graduate	83	37.4
Years lived in the United States	10 or less	53	23.9
	11–20	59	26.6
	More than 20	110	49.5
Currentlywork	Yes	210	94.6
outside home	No	12	5.4
Relationship to family member with dementia	Spouse	9	4.1
	Child orsibling	70	31.5
	Child or sibling in-law	50	22.5
	Grandchild	47	21.2
	Niece or nephew	46	20.7
Family member has a formal dementia diagnosis	Yes	100	45.0
	No	122	55.0
Familymember with dementia lives	Alone	17	7.7
	Currently lives with you	62	27.9
	Lives with someone else	127	57.2
	Lives in nursing home/facility	16	7.2
English spoken at home	Yes	106	60.0
	No	72	40.0
Primary language spoken at home	Hindi	63	28.4
	Punjabi	72	32.4
	Gujrati	30	13.5
	Telugu	16	7.2
	Bengali	9	4.1
	Marathi	8	3.6
	English	12	5.4
	Others	12	5.4
Region	North	166	74.8
	South	55	24.8

**TABLE 2:** Fit indices for short version of Affiliate Stigma Scale confirmatory factor analyses.

Fit index	Acceptable range	Three-factor (22 items)	Interpretation	One-factor (22 items)	Interpretation	One-factor (11 items)	Interpretation

*χ^2^/df*	≤ 3 (acceptable) ≤ 5 (tolerable)	4.60	Tolerable	4.55	Tolerable	2.60	Acceptable
GFI	≥0.90	0.66	Poor fit	0.66	Poor fit	0.91	Good fit
CFI	≥0.95	0.80	Poor fit	0.80	Poor fit	0.95	Good fit
TLI	≥0.95	0.78	Poor fit	0.78	Poor fit	0.95	Good fit
RMSEA	≤0.08	0.12	Poor fit	0.13	Poor fit	0.85	Acceptable
SRMR	≥0.08	0.07	Good Fit	0.05	Good Fit	0.085	Good fit

Abbreviations: *χ^2^*, chi squared; CFI, comparative fit index; df, degrees of freedom ratio; GFI, goodness of fit index; RMSEA, root mean square error of approximation; SRMR, standardized root mean square; TLI, Tucker-Lewis Index.

**TABLE 3:** Descriptives of short version of Affiliate Stigma Scale items.

Total items = 11. *N* = 222 Cronbach’s *α* = 0.93	Mean	Standard deviation	Factor loading	Corrected item-total correlation	Cronbach’s *α* if item deleted

1.1 feel inferior because one of my family members has dementia.	1.83	0.85	0.65	0.591	0.940
2. The behavior of my family member with dementia is embarrassing.	1.86	0.84	0.80	0.760	0.932
3.1 avoid going out with my family member who has dementia.	1.82	0.82	0.80	0.756	0.932
4. People’s attitudes toward me are negative when I am with my family member who has dementia.	1.76	0.74	0.81	0.776	0.931
5. I reduce contact with my friends and relatives because I have a family member with dementia.	1.65	0.74	0.76	0.717	0.934
6. Having a family member with dementia has a negative impact on me.	1.89	0.84	0.81	0.766	0.932
7. When I am with my family member with dementia, I keep a relatively low profile.	1.89	0.84	0.76	0.715	0.934
8. Having a family member with dementia makes me think that I am incompetent compared to other people.	1.58	0.68	0.80	0.746	0.933
9. I reduced interacting with my family member who has dementia.	1.67	0.72	0.80	0.753	0.932
10. Having a family member with dementia makes me think that I am less than others.	1.59	0.68	0.83	0.782	0.932
11. I reduce contact with my neighbors because I have a family member with dementia.	1.65	0.76	0.84	0.793	0.931

**TABLE 4:** Known-groups comparison between demographic characteristics and total score of short version of Affiliate Stigma Scale.

Group	Total score M (SD)	Statistical test and significance	Effect size

**Sex**			
Male	18.23 (7.02)	*t* = −2.762	Cohen’s *d* = −0.304 (small)
Female	20.25 (6.24)	*p* = 0.012*	
**Region**			
North	18.49 (6.44)	*t* = −2.592	Cohen’s *d* = −0.403 (small)
South	21.16 (7.17)	*p* = 0.005*	
**Family relationship**			
Spouse	20.1 (8.7)	*F* = 2.202	Partial *η*^*2*^ = 0.039 (small)
		*p* = 0.070	
Child/sibling	19.7 (7.1)		
In-law	20.0 (5.7)		
Grandchild	19.9 (7.1)		
Niece/nephew	14.8 (6.0)		
**Language**			
Hindi	18.48 (5.93)	*F* = 2.214	Partial *η*^*2*^ = 0.068 (medium)
Punjabi	19.54 (7.93)	*p* = 0.034*	
Gujarati	16.33 (5.17)		
Telugu	19.00 (5.93)		
Bengali	24.33 (5.45)		
Marathi	21.38 (5.32)		
English	22.17 (6.67)		
Others	19.92 (6.40)		

* *p* < 0.05.

** *p* < 0.01.

## Data Availability

The individual-level data supporting this study’s findings are not publicly available due to privacy or ethical restrictions.

## Highlights

- This study validated the 11-item short version of the Affiliate Stigma Scale for Asian Indian dementia family caregivers, demonstrating excellent reliability (Cronbach’s *α* = 0.93) and a unidimensional factor structure.
- The study provides empirical support for a unidimensional representation consistent with the instrument’s original overarching construct, even though it includes three conceptual domains (cognitive, affective, and behavioral).
- Showed the instrument’s ability to detect variations in stigma by sex and region of origin, supporting its utility and cultural relevance.
- Underscored the value of culturally adapted tools for guiding stigma-reduction interventions and informing future research, including cross-cultural applications and translations.

## RESEARCH IN CONTEXT

1. **Systematic review**: We conducted a comprehensive literature review using databases such as PubMed, CINAHL, Google Scholar, and PsycINFO to identify studies on dementia-related stigma, particularly among Asian Indian caregivers. Keywords included “dementia stigma,” “Asian Indian caregivers,” and “Affiliate Stigma Scale.” Relevant articles were analyzed to understand existing knowledge and gaps.
2. **Interpretation**: Our study validated the 11-item short version of the Affiliate Stigma Scale for Asian Indian dementia family caregivers, demonstrating excellent reliability (Cronbach’s *α* = 0.93) and a robust unidimensional factor structure. The findings highlight meaningful variations in stigma by sex and region of origin, supporting its value as a culturally sensitive tool for assessing affiliate stigma among Asian Indian dementia family caregivers.
3. **Future directions**: The availability of a culturally adapted short form of the Affiliate Stigma Scale emphasizes the importance of developing and validating stigma measures tailored to diverse populations. Future research should examine stigma trajectories longitudinally and expand accessibility through translation into additional languages to support use in varied clinical practice and community settings.
