# Supplementary material for: Psychometric evaluation of the Affiliate Stigma Scale for Asian Indian dementia family caregivers living in the United States
Source: Alzheimers Dement Behav Socioecon Aging. Author manuscript; Available in PMC 2026 Mar 10. (PMC12970953; doi:10.1002/bsa3.70047)
Supplement: Affiliate Stigma Scale for Dementia Family Caregivers [file NIHMS2135839-supplement-Affiliate_Stigma_Scale_for_Dementia_Family_Caregivers.docx]

Dementia is a general term for loss of memory, language, problem-solving, and other thinking abilities that is severe enough to interfere with daily life. The most common types of dementia include Alzheimer’s disease, vascular dementia, Pick’s disease (frontotemporal dementia), Lewy body dementia, Parkinson’s disease-related dementia, Huntington’s disease (associated with early-onset dementia), LATE (Limbic-predominant age-related TDP-43 encephalopathy), and mixed dementia.

**Short Version of the Affiliate Stigma Scale for Asian Indian Dementia Family Caregivers**

**Instructions:** Below are some sentences related to your life as a family caregiver of a person living with dementia. There are no right or wrong answers. Please read each sentence carefully, then choose the option that best represents your opinion.

|  | Strongly Disagree | Disagree | Agree | Strongly Agree |
| --- | --- | --- | --- | --- |
| 1. I feel inferior because one of my family members has dementia. | ☐ | ☐ | ☐ | ☐ |
| 1. The behavior of my family member with dementia is embarrassing. | ☐ | ☐ | ☐ | ☐ |
| 1. I avoid going out with my family member who has dementia. | ☐ | ☐ | ☐ | ☐ |
| 1. People’s attitudes towards me are negative when I am with my family member who has dementia. | ☐ | ☐ | ☐ | ☐ |
| 1. I reduce contact with my friends and relatives because I have a family member with dementia. | ☐ | ☐ | ☐ | ☐ |
| 1. Having a family member with dementia has   a negative impact on me. | ☐ | ☐ | ☐ | ☐ |
| 1. When I am with my family member with dementia, I keep a relatively low profile. | ☐ | ☐ | ☐ | ☐ |
| 1. Having a family member with dementia makes me think that I am incompetent compared to other people. | ☐ | ☐ | ☐ | ☐ |
| 1. I reduced interacting with my family member who has dementia. | ☐ | ☐ | ☐ | ☐ |
| 1. Having a family member with dementia makes me think that I am less than others. | ☐ | ☐ | ☐ | ☐ |
| 1. I reduce contact with my neighbors because   I have a family member with dementia. | ☐ | ☐ | ☐ | ☐ |

Published in: Wadhawan A, Btoush R, Zha P, Jarrín OF. Psychometric evaluation of the Affiliate Stigma Scale for Asian Indian dementia family caregivers living in the United States. *Alzheimer's & Dementia: Behavior & Socioeconomics of Aging*. 2025; 1(4):e70047. https://doi.org/10.1002/bsa3.70047
