# Supplementary material for: Psychometric evaluation of the Affiliate Stigma Scale for Asian Indian dementia family caregivers living in the United States
Source: Alzheimers Dement Behav Socioecon Aging. Author manuscript; Available in PMC 2026 Mar 10. (PMC12970953; doi:10.1002/bsa3.70047)
Supplement: Recruitment Flyer [file NIHMS2135839-supplement-Recruitment_Flyer.pdf]

RUTGERS HEALTH
School of Nursing

WHO: Asian Indian Dementia Caregivers in the United States!

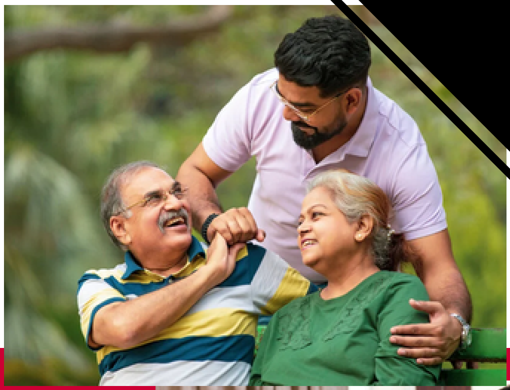

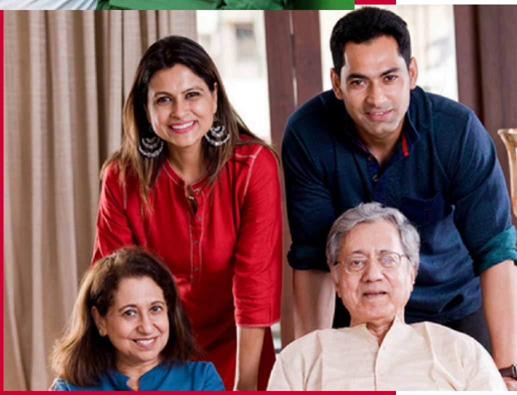

Why ?

Participate in the Study

- **Help in establishing a reliable tool for measuring affiliate stigma**
- **Contribute to research that can improve support for caregivers**
- **Gain insights that could enhance caregiving experiences**

How: Complete the Questionnaire

Eligibility Criteria

***must fit all of the below**

- Adults 18 + years
- Indian origin
- Dementia Caregivers
- Living in the United States

Scan QR Code:

Please Contact

For More Information:

Anju Wadhawan, MSN, MHA, RN, PhD student,
aw933@sn.rutgers.edu
Olga Jarrin, PhD, RN, FAAN, olga.jarrin@rutgers.edu

RUTGERS | eIRB
APPROVED
IRB ID: Pro2024001049
Approval Date: 9/4/2024
Expiration Date: 12/31/2499
