# Supplementary material for: Psychometric evaluation of the Affiliate Stigma Scale for Asian Indian dementia family caregivers living in the United States
Source: Alzheimers Dement Behav Socioecon Aging. Author manuscript; Available in PMC 2026 Mar 10. (PMC12970953; doi:10.1002/bsa3.70047)
Supplement: Table s1 [file NIHMS2135839-supplement-Table_s1.docx]

Table S1. *Demographic Characteristics of Study Sample and 11-item Asian Indian Dementia Family Caregiver Affiliate Stigma Scale Score*

|  | **Category** | **n** | **Valid %** | **Mean (SD)** | **Median (IQR)** | **Min-Max** | **Statistic** |
| --- | --- | --- | --- | --- | --- | --- | --- |
|  | Total Sample | 222 | 100% | 19.2 (6.7) | 19 (12-24) | 11-42 |  |
| Age in years | < 45 | 85 | 38.3% | 19.2 (6.9) | 19 (12-24) | 11-37 | *F* = 0.046  *p* = 0.955 |
|  | 45-54 | 77 | 34.7% | 19.4 (6.5) | 19 (13-24) | 11-36 |  |
|  | 55+ | 60 | 27.0% | 19.0 (6.9) | 19.5 (12-23) | 11-42 |  |
| Sex | Male | 117 | 52.7% | 18.2 (7.0) | 17 (11-23) | 11-42 | *t* = -2.762  *p* = 0.012 |
|  | Female | 105 | 47.3% | 20.3 (6.2) | 22 (15-24.5) | 11-36 |  |
| Marital Status | Married/domestic partnership | 180 | 81.1% | 19.0 (6.6) | 19 (12-23) | 11-42 | *F* = 0.257  *p* = 0.905 |
|  | Single (never married) | 32 | 14.4% | 19.7 (7.7) | 20.5 (11-24) | 11-37 |  |
|  | Divorced / Separated | 6 | 2.7% | 20.2 (6.0) | 20.5 (14-24) | 13-30 |  |
|  | Widowed | 4 | 1.8% | 21.5 (3.3) | 20.5 (19-25) | 19-26 |  |
| Education Level | High School or Trade school | 45 | 20.3% | 18.7 (6.8) | 19 (11.5-23) | 11-37 | *F* = 0.247  *p* = 0.781 |
|  | Bachelor’s degree | 94 | 42.3% | 19.5 (6.7) | 20 (13-24) | 11-37 |  |
|  | Graduate | 83 | 37.4% | 19.1 (6.8) | 20 (12-24) | 11-42 |  |
| Years lived in the U.S. | 10 or less | 54 | 23.9% | 17.9 (6.9) | 17 (11-23) | 11-37 | *F* = 1.925  *p* = 0.148 |
|  | 11 – 20 | 59 | 26.6% | 18.9 (6.1) | 19 (13-23) | 11-33 |  |
|  | More than 20 | 110 | 49.5% | 20.1 (6.9) | 21 (13-24) | 11-42 |  |
| Work outside home | Yes | 210 | 94.6% | 19.3 (6.8) | 19 (12-23) | 11-42 | *t* = -0.629  *p* = 0.265 |
|  | No | 12 | 5.4% | 18.0 (5.8) | 19.5 (16-23) | 11-26 |  |
| Relationship to family member with dementia | Spouse | 9 | 4.1% | 20.1 (8.7) | 19 (13-26) | 11-37 | *F* = 2.202  *p* = 0.070 |
|  | Child or sibling | 70 | 31.5% | 19.7 (7.1) | 20.5 (12-24) | 11-42 |  |
|  | Child or sibling in-law | 50 | 22.5% | 20.0 (5.7) | 21 (16-23) | 11-33 |  |
|  | Grandchild | 47 | 21.2% | 19.9 (7.1) | 20 (12-25) | 11-33 |  |
|  | Niece or Nephew | 46 | 20.7% | 14.8 (6.0) | 14.5 (11-22) | 11-30 |  |
| Family member has a dementia diagnosis | Yes  No | 100  122 | 45%  55% | 20.0 (7.3)  18.6 (6.2) | 20.5 (13-25)  19.0 (12-23) | 11-42  11-33 | *t* = -1.551  *p* = 0.061 |
| Family member with dementia lives | Lives alone | 17 | 7.7% | 20.9 (7.3) | 22 (13.5-24) | 11-37 | *F* = 1.627  *p* = 0.169 |
|  | Currently lives with you | 62 | 27.9% | 19.9 (7.1) | 20.5 (13-24) | 11-42 |  |
|  | Lives with someone else | 127 | 57.2% | 18.3 (6.5) | 17 (12-23) | 11-32 |  |
|  | Lives in nursing home | 16 | 7.2% | 21.6 (6.0) | 22 (17-26) | 11-31 |  |

| English spoken at home | Yes | 106 | 60% | 19.2 (6.9) | 18.5 (12-24) | 11-37 | *t* = -0.662  *p* = 0.254 |
| --- | --- | --- | --- | --- | --- | --- | --- |
|  | No | 72 | 40% | 18.5 (6.3) | 19 (12-23) | 11-42 |  |
| Primary language spoken at home | Hindi | 63 | 28.4% | 18.5 (5.9) | 19 (12-23) | 11-37 | *F* = 2.214  *p* = 0.034 |
|  | Punjabi | 72 | 32.4% | 19.5 (7.9) | 17.5 (11-26) | 11-42 |  |
|  | Gujrati | 30 | 13.5% | 16.3 (5.2) | 13.0 (12-22) | 11-27 |  |
|  | Telugu | 16 | 7.2% | 19.0 (5.9) | 20 (12.5-24) | 11-29 |  |
|  | Bengali | 9 | 4.1% | 24.3 (5.5) | 24 (20.5-30) | 15-31 |  |
|  | Marathi | 8 | 3.6% | 21.4 (5.3) | 23.5 (17-25) | 11-27 |  |
|  | English | 12 | 5.4% | 22.2 (6.7) | 22.5 (17-25) | 11-36 |  |
|  | Other (e.g.,) | 12 | 5.4% | 19.9 (6.4) | 22 (13-25) | 11-29 |  |
| Region of family origin in India | North | 166 | 74.8% | 18.5 (6.4) | 18 (12-23) | 11-42 | *t* = -2.592  *p* = 0.005  *F*(1, 217) = 7.54 *p* = 0.007 |
|  | Male | 82 |  | 16.7 (6.2) | 14 (11-22) | 11-42 |  |
|  | Female | 84 |  | 20.3 (6.2) | 21.5 (15-24) | 11-36 |  |
|  | South | 55 | 24.8% | 21.2 (7.2) | 22 (13-25) | 11-37 |  |
|  | Male | 35 |  | 21.9 (7.5) | 22 (14-28) | 11-37 |  |
|  | Female | 20 |  | 19.9 (6.5) | 21.5 (13-25) | 11-33 |  |
